# Active smoking and risk of breast cancer in a Danish nurse cohort study

**DOI:** 10.1186/s12885-017-3546-4

**Published:** 2017-08-22

**Authors:** Zorana Jovanovic Andersen, Jeanette Therming Jørgensen, Randi Grøn, Elvira Vaclavik Brauner, Elsebeth Lynge

**Affiliations:** 1 Centre for Epidemiology and Screening, Department of Public Health, University of Copenhagen, Øster Farimagsgade 5, 1014 Copenhagen, Denmark; 2 Department of Occupational and Environmental Medicine, Bispebjerg - Frederiksberg Hospital, University of Copenhagen, Bispebjerg Bakke 23, Building 20C, 2400 Copenhagen, Denmark; 3 Biomarkers and Clinical Research in Eating Disorders, Ballerup Center for Mental Health Services, Capitol Region of Denmark, Rigshospitalt-Ballerup, Denmark

**Keywords:** Tobacco smoking, Active smoking, Breast cancer, Cohort

## Abstract

**Background:**

No scientific consensus has been reached on whether active tobacco smoking causes breast cancer. We examine the association between active smoking and breast cancer risk in Denmark, which has some of the highest smoking and breast cancer rates in women worldwide.

**Methods:**

We used the data from a nationwide Danish Nurse Cohort on 21,867 female nurses (age > 44 years) who at recruitment in 1993 or 1999 reported information on smoking status, onset, duration, and intensity, as well as breast cancer risk factors. We obtained data on incidence of breast cancer from Danish Cancer Registry until 2013, and used Cox regression models to analyze the association between smoking and breast cancer.

**Results:**

Of 21,831 women (mean age 53.2 years) 1162 developed breast cancer during 15.7 years of follow-up. 33.7% of nurses were current and 30.0% former smokers at cohort baseline. Compared to never smokers, we found increased risk of breast cancer of 18% in ever (hazard ratio and 95% confidence interval: 1.18; 1.04–1.34) and 27% in current (1.27; 1.11–1.46) smokers. We detected a dose-response relationship with smoking intensity with the highest breast cancer risk in women smoking >15 g/day (1.31; 1.11–1.56) or >20 pack-years (1.32; 1.12–1.55). Parous women who smoked heavily (>10 pack-years) before first childbirth had the highest risk of breast cancer (1.58; 1.20–2.10). Association between smoking and breast cancer was not modified by menopausal status, obesity, alcohol or hormone therapy use, and seemed to be limited to the estrogen receptor positive breast cancer subtype.

**Conclusions:**

Active smoking increases risk of breast cancer, with smoking before first birth being the most relevant exposure window.

## Background

Tobacco smoke is the leading cause of cancer worldwide and contains over 4000 known carcinogenic substances. [1] No scientific consensus has been reached on whether active tobacco smoking causes breast cancer, despite 25 years of debate and over 150 epidemiological studies. [2, 3] Smoking has been suggested to have an anti-estrogenic effect [4] and should thus be expected to protect against breast cancer in post-menopausal women. However, a meta-analysis of 53 epidemiological studies found no impact of smoking on the risk of breast cancer. [5] Early studies on smoking and breast cancer risk have however been criticized for their crude definitions of smoking and lack of information on intensity, duration, and onset, or use of hospital based controls in case-control studies, which may explain the null associations reported. [5]

Recent large prospective cohort studies [6–13] with detailed data on active smoking consistently report an increased breast cancer risk associated with longer duration and higher intensity of smoking, and indicating that smoking early in life, before first childbirth, is the most relevant exposure window. [6, 7, 9, 11] Still, some inconsistencies exist as not all recent studies linked active smoking to breast cancer. [14, 15] The latest reports from the International Agency for Research on Cancer (IARC) [1] and the US Surgeon General [3] conclude that there was suggestive, but insufficient, evidence to infer a causal relationship between active smoking and breast cancer, [3] calling for more data.

In Denmark, with some of the highest smoking prevalence and breast cancer incidence in the world, the impact of smoking on breast cancer has been debated due to conflicting results from the early Danish studies. [16, 17] More recently, no association between smoking and breast cancer was detected in 28,000 women from Danish Diet, Cancer and Health cohort which formed part of the large European Prospective investigation into Cancer and Nutrition (EPIC) study. [11] In the EPIC study, the observed overall positive association between smoking and breast cancer was driven by data from the other non-Danish EPIC cohorts. [11] On this basis, we investigated the association between active smoking and breast cancer risk in members of the Danish Nurse Cohort, which is a large, nationwide cohort of female nurses older than 44 years.

## Methods

### The Danish nurse cohort

The Danish Nurse Cohort [18] was inspired by the American Nurses’ Health Study to initially investigate the health effects of hormone therapy (HT) in a European population. The cohort was initiated in 1993 by sending a questionnaire to 23,170 female Danish nurses (> 44 years), members of the Danish Nursing Organization, which included 95% of all nurses in Denmark. In total, 19,898 (86%) nurses replied, and the cohort was reinvestigated in 1999, including an additional 10,534 nurses (who turned 44 years in the period 1993–99), of whom 8833 (84%) replied. Nurses filled out the questionnaire at recruitment on working conditions, weight and height, lifestyle (diet, active smoking, alcohol consumption, and leisure time physical activity), parity, age at first birth, age of menarche and menopause, and use of oral contraceptives (OC) and HT. We utilized baseline information from 1993 (19,898) or 1999 (8833) for 28,731 female nurses. Using a unique identification number we linked the cohort participants to Civil Registration System [19] to obtain vital status information at 31st December 2012 (active, date of death or emigration).

### Active tobacco smoking

Data on active tobacco smoking were obtained from the baseline questionnaire in 1993 or 1999, and included questions on smoking status (never/former/current), smoking duration (years), age at smoking onset (years), average number of cigarettes, cheerots, and cigars smoked per day, and on smoking a pipe (yes/no) in ever smokers. Based on this information we calculated smoking intensity in g/day by equating a cigarette to 1 g, a cheroot to 3 g, and a cigar to 4.5 g of tobacco, and pack-years of smoking by multiplying the number of packs per day (1 pack = 20 g) and the number of years smoked. We defined onset of smoking before and after 1st birth in parous women and age of 21 years (mean age of smoking initiation in the cohort) in nulliparous women. Pack-years of smoking before 1st childbirth, between 1st childbirth and menopause, and after menopause was calculated from information on age of smoking onset, age at 1st birth, and age at menopause in parous postmenopausal women. No information was collected on passive smoking in the Danish Nurse Cohort.

### Breast cancer definition

We linked the records of 28,731 nurses using unique identification number to the Danish Cancer Register [20] to extract all cancer diagnoses until 2013. First, we extracted data for nurses with diagnoses for any (other than non-melanoma skin cancer) cancer before baseline (1st April 1993 or 1st April 1999), these nurses were excluded from the analyses. Secondly, among nurses without prior cancer, we extracted primary invasive breast cancer diagnoses (ICD-10 codes C50), as the main outcome, and any other cancer (other than non-melanoma skin cancer) between cohort baseline (1st April 1993 or 1st April 1999) and 31st December 2012. Furthermore, we extracted data on breast cancer subtype by estrogen receptor (ER) and progesterone receptor (PR) status from the clinical database of the Danish Breast Cancer Cooperative Group. [21]

### Statistical analyses

We used Cox proportional hazards regression with age as the underlying time, to investigate the association between smoking and breast cancer in a crude model (age adjusted as age is underlying time scale), and in a fully adjusted model, adjusted for age at the time of recruitment, birth cohort (1990–1934; 1935–1944; 1945–1949; 1950–1955), Body Mass Index (BMI) (<18.5 kg/m^2^; 18.5–25 kg/m^2^; 25–30 kg/m^2^; ≥30 kg/m^2^), alcohol use (none; moderate (1–14 drinks/week); heavy (>15 drinks/week)), leisure time physical activity (low; medium; high), night shift work (yes; no), age at menarche (years), parity (yes; no), number of children, age at first birth (years), menopausal status (yes; no), HT use (never, ever), and OC use (never; ever). The follow-up started on the cohort baseline date (1st April 1993 or 1st April 1999) and ended at the date of breast cancer (event) or other cancer diagnoses (except non-melanoma skin cancer), death, emigration, or December 31, 2012 (censoring), whichever came first. We evaluated the effect of active smoking status, duration, intensity, and onset in separate models. We performed tests for trend by using the ordered category, including the reference as a continuous variable in the Cox model. We checked for the proportional hazards assumption for all smoking variables and confounders based on scaled Schoenfeld residuals. [22] The effect modification of an association between smoking and breast cancer by menopausal status, obesity, alcohol use, and HT use was evaluated by introducing interaction terms into the Cox model, and tested by the Wald test. Finally, separate models were fit for subtypes of breast cancer according to ER status (ER+; ER-) and ER status combined with PR status (ER+/PR+; ER+/PR-; ER−/PR-; ER−/PR+) as outcomes. Results were presented as hazard ratios (HRs) and 95% confidence intervals (CI). Analyses were performed in Stata 11.2.

The study was entirely based on a data from registers and approved by the Danish Data Inspection Agency, which by Danish law serving as ethical approval of register-based research. Thus, no contact has been taken with participating women, relatives or their practicing doctors, and no consent was needed.

## Results

Of the total 28,731 nurses in the Danish Nurse Cohort, we excluded 4 due to inactive (emigrated) vital status and 1924 with cancer diagnosis before cohort baseline, and 4972 with missing information on one or more covariates. Of the 21,831 nurses in the main analyses 1162 developed breast cancer during the mean follow-up of 15.7 years or 342,538 person-years, with an incidence rate of 339 per 100,000 person-years.

The mean age at baseline was 53.2 years, 56.7% of the women were postmenopausal, 14.5% nulliparous, and the mean age at 1st childbirth for parous women was 25.9 years (Table 1). Compared with women who remained free of breast cancer, those who developed the cancer were more likely to be nulliparious, postmenopausal, obese, heavy alcohol drinkers, slightly physically active and HT users, but less likely to work night shifts, and use OC.

**Table 1:** Description of the Danish Nurse Cohort (*n* = 21,831) at the time of recruitment in 1993 or 1999 and by breast cancer status during follow-up until 2013

	Total *N* = 21,831	Breast Cancer *N* = 1162	No Breast Cancer *N* = 20,669
Age			
Mean (SD) age at baseline (years)	53.2 (8.0)	53.7 (7.6)	53.2 (8.1)
Birth Cohort			
Born 1900–1934, n (%)	5179 (23.7)	295 (25.4)	4884 (23.6)
Born 1935–1944, n (%)	6707 (30.7)	428 (36.8)	6279 (30.4)
Born 1945–1949, n (%)	4564 (20.9)	244 (21.0)	4320 (20.9)
Born 1950–1955, n (%)	5381 (24.6)	195 (16.8)	5186 (25.1)
Reproductive Factors			
Mean (SD) age at menarche	13.5 (1.5)	13.5 (1.5)	13.5 (1.5)
Nulliparous, n (%)	3170 (14.5)	189 (16.3)	2981 (14.4)
Mean (SD) number of children in parous women	2.3 (0.9)	2.3 (0.9)	2.3 (0.9)
Mean (SD) age at first birth in parous women	25.9 (4.0)	25.9 (4.0)	26.3 (4.1)
Postmenopausal, n (%)	12,376 (56.7)	696 (59.9)	11,680 (56.5)
Body Mass Index (BMI)			
Mean (SD) BMI (kg/m^2^)	23.7 (3.5)	23.8 (3.5)	23.7 (3.5)
BMI < 18.5 kg/m^2^, n (%)	529 (2.4)	22 (1.9)	507 (2.5)
BMI 18.5–24.9 kg/m^2^, n (%)	15,070 (69.0)	819 (70.5)	14,251 (68.9)
BMI 25–29.9 kg/m^2^, n (%)	4979 (22.8)	245 (21.1)	4734 (22.9)
BMI ≥ 30 kg/m^2^, n (%)	1253 (5.7)	76 (6.5)	1177 (5.7)
Alcohol consumption			
Does not drink alcohol, n (%)	3320 (15.2)	183 (15.7)	3137 (15.2)
Moderate drinker (1–14 drinks/week), n (%)	13,533 (62.0)	670 (57.7)	12,863 (62.2)
Heavy drinker (> 14 drinks/week), n (%)	4978 (22.8)	309 (26.6)	4669 (22.6)
Leisure time physical activity			
Low physical activity, n (%)	1428 (6.5)	79 (6.8)	1349 (6.5)
Medium physical activity, n (%)	5905 (27.0)	298 (25.6)	5607 (27.1)
High physical activity, n (%)	14,498 (66.4)	785 (67.6)	13,713 (66.3)
Shift Work			
Working night shifts, n (%)	967 (5.5)	45 (4.9)	922 (5.5)
Hormone Use			
Ever used oral contraceptives, n (%)	12,701 (58.2)	663 (57.1)	12,038 (58.2)
Ever used hormone therapy, n (%)	5951 (27.3)	407 (35.0)	5544 (26.8)
Smoking Status			
Never smoker, n (%)	7923 (36.3)	379 (32.6)	7544 (36.5)
Former smoker, n (%)	6557 (30.0)	338 (29.1)	6219 (30.1)
Current smoker, n (%)	7351 (33.7)	445 (38.3)	6906 (33.4)
Ever smoker, n (%)	13,908 (63.7)	783 (67.4)	13,125 (63.5)
Smoking Intensity			
Mean (SD) smoking duration^a^ (years)	23.3 (12.2)	23.9 (11.7)	23.3 (12.2)
Mean (SD) smoking intensity^a^ (g/day)	12.4 (8.3)	12.7 (8.0)	12.4 (8.3)
Mean (SD) pack-years^a^	16.0 (14.8)	16.6 (14.8)	15.9 (14.8)
Initiation of smoking			
Mean age (SD) at smoking initiation^a^ (years)	20.4 (5.5)	20.7 (5.7)	20.4 (5.5)
Initiation of smoking in 18,661 parous women			
Parous never smoker	6938 (37.2)	326 (33.5)	6612 (37.4)
Parous ever smoker	11,723 (62.8)	647 (66.5)	11,076 (62.6)
Parous/around 1st birth or later, n (%)	1457 (6.7)	88 (7.6)	1369 (6.6)
Parous/before 1st childbirth, ≤ 10 pack-years, n (%)	9367 (42.9)	493 (42.4)	8874 (42.9)
Parous/before 1st childbirth, > 10 pack-years, n (%)	899 (4.1)	66 (5.7)	833 (4.0)
Initiation of smoking in 3170 nulliparous women			
Nulliparous never smoker	985 (31.1)	53 (28.0)	932 (31.3)
Nulliparous ever smoker	2185 (68.9)	136 (72.0)	2049 (68.7)
Nulliparous, before age 21^b^ years, n (%)	1264 (5.8)	74 (6.4)	1190 (5.8)
Nulliparous, age 21 years or later, n (%)	921 (4.2)	62 (5.3)	859 (4.2)
Initiation of smoking in 12,376 postmenopausal women			
Postmenopausal never smoker, n (%)	3988 (32.2)	202 (29.0)	3786 (32.4)
Before menopause, n (%)	8301 (67.1)	488 (70.1)	7813 (66.9)
After menopause, n (%)	87 (0.7)	6 (0.9)	81 (0.7)

*SD* standard deviation; ^a^in ever smokers. ^b^21 is mean age of smoking initiation in the cohort

The majority of nurses (63.7%) were ever smokers at cohort baseline, (33.7% current, 30.0% previous) whilst 36.3% never smokers. 38.3% of women who developed breast cancer were current smokers at baseline, as compared to 33.4% of women who were free of breast cancer (Table 1). Mean duration of smoking in ever smokers was 23.3 years, mean intensity 12.4 g/day or 16.0 pack-years, and mean age at smoking initiation 20.4 years. Smoking duration and intensity were higher in nurses who developed breast cancer than in those who were free of breast cancer. 62.8% of parous women and 68.9% of nulliparous women were ever smokers. The majority of women started smoking early, before 1st childbirth or before age 21 (nulliparous women).

Whilst smoking rates and smoking intensity were lower in younger, as compared to older birth cohorts, the age at smoking initiation decreased, from 22.6 years in women born before 1935, to 17.8 in women born 1950–55 (Table 2). Accordingly, number of women smoking before 1st childbirth increased. Notably, also the use of HT and alcohol was higher in younger than in older birth cohorts.

**Table 2:** Description of the Danish Nurse Cohort (*n* = 21,831) at the time of recruitment in 1993 or 1993 and by birth cohort

Birth Cohort	1900–1934 *N* = 5179	1935–1944 *N* = 6707	1945–1949 *N* = 4564	1950–1955 *N* = 5381
Age				
Mean (SD) age at baseline (years)	65.3 (5.8)	53.1 (3.0)	47.7 (1.9)	46.4 (1.5)
Reproductive Factors				
Mean (SD) age at menarche	14.0 (1.5)	13.6 (1.6)	13.4 (1.5)	13.2 (1.5)
Nulliparous, n (%)	1401 (27.1)	764 (11.4)	431 (9.4)	574 (10.7)
Mean (SD) number of children in parous women	2.5 (1.0)	2.4 (0.9)	2.3 (0.8)	2.3 (0.8)
Mean (SD) age at first birth in parous women	27.9 (4.1)	25.7 (3.6)	25.0 (3.6)	25.6 (4.2)
Postmenopausal, n (%)	5170 (99.8)	5060 (75.4)	1240 (27.2)	906 (16.8)
Body Mass Index (BMI)				
Mean (SD) BMI (kg/m^2^)	23.8 (3.5)	23.7 (3.4)	23.5 (3.4)	23.8 (3.6)
BMI < 18.5 kg/m^2^, n (%)	199 (3.8)	148 (2.2)	100 (2.2)	82 (1.5)
BMI 18.5–24.9 kg/m^2^, n (%)	3368 (65.0)	4657 (69.4)	3293 (72.2)	3752 (69.7)
BMI 25–29.9 kg/m^2^, n (%)	1315 (25.4)	1527 (22.8)	944 (20.7)	1193 (22.2)
BMI ≥ 30 kg/m^2^, n (%)	297 (5.7)	375 (5.6)	227 (5.0)	354 (6.6)
Alcohol consumption				
Does not drink alcohol, n (%)	1278 (24.7)	980 (14.6)	516 (11.3)	546 (10.1)
Moderate drinker (1–14 drinks/week), n (%)	2900 (56.0)	4177 (62.3)	2923 (64.0)	3533 (65.7)
Heavy drinker (> 14 drinks/week), n (%)	1001 (19.3)	1550 (23.1)	1125 (24.6)	1302 (24.2)
Leisure time physical activity				
Low physical activity, n (%)	467 (9.0)	392 (5.8)	250 (5.5)	319 (5.9)
Medium physical activity, n (%)	1090 (21.0)	1809 (27.0)	1406 (30.8)	1600 (29.7)
High physical activity, n (%)	3622 (69.9)	4506 (67.2)	2908 (63.7)	3462 (64.3)
Shift Work				
Working night shifts, n (%)	191 (12.5)	453 (7.4)	178 (4.0)	145 (2.7)
Hormone Use				
Ever used oral contraceptives, n (%)	2082 (40.2)	2490 (37.1)	779 (17.1)	600 (11.2)
Ever used hormone therapy, n (%)	1143 (22.1)	3544 (52.8)	3462 (75.9)	4552 (84.6)
Smoking status				
Never smoker, n (%)	1481 (28.6)	2487 (37.1)	1840 (40.3)	2115 (39.3)
Former smoker, n (%)	1812 (35.0)	1716 (25.6)	1242 (27.2)	1787 (33.2)
Current smoker, n (%)	1886 (36.4)	2504 (37.3)	1482 (32.5)	1479 (27.5)
Ever smoker, n (%)	3698 (71.4)	4220 (62.9)	2724 (59.7)	3266 (60.7)
Smoking Intensity				
Mean (SD) smoking duration^a^ (years)	29.4 (14.5)	23.4 (11.3)	19.8 (9.5)	19.4 (9.2)
Mean (SD) smoking intensity^a^ (g/day)	12.1 (9.0)	13.0 (8.8)	12.5 (7.5)	12.0 (7.5)
Mean (SD) pack-years^a^	19.5 (18.6)	16.9 (14.9)	13.5 (11.1)	12.8 (11.0)
Initiation of smoking				
Mean age (SD) at smoking initiation^a^ (years)	22.6 (6.9)	21.1 (5.3)	19.6 (3.9)	17.8 (3.3)
Initiation of smoking in 18,661 parous women				
Parous never smoker, n (%)	1078 (28.5)	2246 (37.8)	1684 (40.8)	1930 (40.2)
Parous ever smoker, n (%)	2700 (71.5)	3697 (62.2)	2449 (59.3)	2877 (59.9)
Parous/around 1st birth or later, n (%)	430 (8.3)	632 (9.4)	259 (5.7)	136 (2.5)
Parous/before 1st childbirth, ≤ 10 pack-years, n (%)	2047 (39.5)	2824 (42.1)	2037 (44.6)	2459 (45.7)
Parous/before 1st childbirth, > 10 pack-years, n (%)	223 (4.3)	241 (3.6)	153 (3.4)	282 (5.2)
Initiation of smoking in 3170 nulliparous women				
Nulliparous never smoker, n (%)	403 (28.8)	241 (31.5)	156 (36.2)	185 (32.2)
Nulliparous ever smoker, n (%)	998 (71.2)	523 (68.5)	275 (63.8)	389 (67.8)
Nulliparous, before age 21^b^ years, n (%)	442 (8.5)	295 (4.4)	187 (4.1)	340 (6.3)
Nulliparous, age 21 years or later, n (%)	556 (10.7)	228 (3.4)	88 (1.9)	49 (0.9)
Initiation of smoking in 12,376 postmenopausal women				
Postmenopausal never smoker, n (%)	1477 (28.6)	1757 (34.7)	454 (36.6)	300 (33.1)
Postmenopausal ever smoker, n (%)	3693 (71.4)	3303 (65.3)	786 (63.4)	606 (66.9)
Before menopause, n (%)	3633 (70.3)	3281 (64.8)	782 (63.1)	605 (66.8)
After menopause, n (%)	60 (1.2)	22 (0.4)	4 (0.3)	1 (0.1)

*SD* standard deviation; ^a^in ever smokers. ^b^21 is mean age of smoking initiation in the cohort

Compared to never smokers, we found an increased risk of breast cancer in ever (HR: 1.18; 95% CI: 1.04–1.34) smokers, strongest in current (1.27; 1.11–1.46), and weaker in previous (1.08; 0.94–1.26) smokers (Table 3). We found a statistically significant dose-response association with increasing smoking duration and intensity, with highest risk of breast cancer observed in women smoking 21–20 years (1.24; 1.06–1.46) and >30 years (1.21; 1.01–1.46), 11–15 g/day (1.22; 1.02–1.46) and >15 g/day (1.31; 1.11–1.56), and >20 pack-years (1.32; 1.12–1.55). Compared to parous never smokers, the risk of breast cancer seemed weaker in parous ever smokers who started smoking before (1.17; 1.02–1.34, results not shown) than after (1.28; 1.00–1.62) 1st childbirth. However, when accounting for smoking intensity, we found the strongest association among parous women who smoked heavily (>10 pack-years) before 1st childbirth (1.58; 1.20–2.10) and weak in those who smoked ≤10 pack-years (1.13; 0.98–1.31). Nulliparous ever smokers had also increased risk of breast cancer, similar to that observed in parous women (1.21; 0.88–1.67, results not shown), and slightly higher with late (≥ age 21) (1.29; 0.89–1.88) than with early (< 21 years) (1.10; 0.76–1.58) onset of smoking. When limiting analyses to the 12,376 women who were postmenopausal at the time of recruitment, we found that both women who started smoking before (1.20; 1.01–1.41) and after (1.66; 0.74–3.75) menopause had increased risk of breast cancer. Finally, when considering smoking intensity in different periods of life related to 1st childbirth and menopause among 8347 parous postmenopausal women, the strongest effect of smoking on breast cancer was observed with smoking before 1st childbirth (11–20 pack-years: 2.16; 1.45–3.20), and a weaker, but still strong effect was seen for smoking between 1st childbirth and menopause (> 20 pack-years: 1.84; 1.34–2.53), while the weakest effects was seen for smoking after menopause (> 20 pack-years: 1.29; 0.70–4.98).

**Table 3:** Association between active smoking and breast cancer in 21,831 women in the Danish Nurse Cohort

	*N*	Person-years	No. of cases	Age adjusted	Fully^a^ adjusted
				HR (95% CI)	HR (95% CI)
Never smoked (ref.)	7923	126,950	379	1.00	1.00
Former smoker	6557	102,190	338	1.11 (0.96–1.29)	1.08 (0.94–1.26)
Current smoker	7351	113,400	445	1.32 (1.15–1.51)	1.27 (1.11–1.46)
Ever smokers	13,908	215,589	783	1.22 (1.08–1.38)	1.18 (1.04–1.34)
Smoking duration among ever smokers					
≤ 10 years	2925	47,335	156	1.10 (0.92–1.33)	1.10 (0.91–1.33)
11–20 years	3175	50,151	175	1.17 (0.98–1,40)	1.15 (0.96–1.38)
21–30 years	4456	68,818	259	1.26 (1.08–1.48)	1.24 (1.06–1.46)
> 30 years	3295	49,285	193	1.32 (1.11–1.57)	1.21 (1.01–1.46)
*p-value for trend*					*0.004*
Every increase of 10 years				1.08 (1.04–1.12)	1.06 (1.02–1-11)
Smoking (Tobacco) Intensity among ever smokers					
> 6 g/day	3098	49,245	161	1.10 (0.91–1.32)	1.07 (0.89–1.29)
6–10 g/day	4317	67,410	235	1.17 (0.99–1.38)	1.14 (0.97–1.34)
11–15 g/day	3050	46,962	175	1.25 (1.05–1.50)	1.22 (1.02–1.46)
> 15 g/day	3443	51,973	212	1.37 (1.16–1.62)	1.31 (1.11–1.56)
*p-value for trend*					*0.001*
Every increase of 10 g/day				1.05 (0.97–1.14)	1.04 (0.95–1.13)
Lifetime pack-years					
≤ 10 pack-years	6082	96,640	327	1.13 (0.98–1.31)	1.12 (0.97–1.30)
11–20 pack-years	3536	54,993	192	1.17 (0.99–1.39)	1.14 (0.96–1.36)
> 20 pack-years	4290	63,956	264	1.39 (1.19–1.63)	1.32 (1.12–1.55)
*p-value for trend*					*0.002*
Every increase of 20 pack-years				1.10 (1.01–1.20)	1.07 (0.98–1.18)
Initiation of smoking in 18,661 parous women					
Parous never smoker (ref.)	6938	111,811	326	1.00	1.00
Parous, around first birth or later	1457	23,553	88	1.28 (1.01–1.62)	1.28 (1.00–1.62)
Parous, before first childbirth, ≤ 10 pack-years	9367	146,965	493	1.15 (1.00–1.33)	1.13 (0.98–1.31)
Parous, before first childbirth, > 10 pack-years	899	12,901	66	1.77 (1.36–2-31)	1.58 (1.20–2.10)
Initiation of smoking in 3170 nulliparous women					
Nulliparous never smoker (ref.)	985	15,138	53	1.00	1.00
Nulliparous, before age 21^b^ years	1264	18,737	74	1.14 (0.80–1.62)	1.10 (0.76–1.58)
Nulliparous, age 21 years or later	921	13,434	62	1.31 (0.91–1.90)	1.29 (0.89–1.88)
Initiation of smoking in 12,376 postmenopausal women					
Postmenopausal never smoker (ref.)	3988	66,617	202	1.00	1.00
Before menopause	8301	130,987	488	1.23 (1.05–1.45)	1.20 (1.01–1.41)
After menopause	87	1252	6	1.60 (0.71–3.59)	1.66 (0.74–3.75)
Pack-years in 8347 parous postmenopausal women^c^					
Parous postmenopausal never smoker (ref.)	2712		129	1.00	1.00
Pack-years before 1st childbirth^d^					
≤ 10 pack-years	4349	70,627	227	1.16 (0.93–1.44)	1.14 (0.92–1.42)
11–20 pack-years	366	5233	34	2.36 (1.62–3.45)	2.16 (1.45–3.20)
> 20 pack-years	61	854	4	1.69 (0.63–4.59)	1.38 (0.50–3.78)
*p-value for trend*					*0.006*
Pack-years from 1st childbirth until menopause^d^					
≤ 10 pack-years	2572	42,484	128	1.08 (0.85–1.38)	1.03 (0.81–1.33)
11–20 pack-years	1606	25,391	79	1.12 (0.85–1.49)	1.13 (0.85–1.50)
> 20 pack-years	817	12,471	60	1.74 (1.28–2.36)	1.84 (1.34–2.53)
*p-value for trend*					*0.029*
Pack-years after menopause^d^					
≤ 10 pack-years	2294	37,270	124	1.20 (0.94–1.53)	1.19 (0.93–1.53)
11–20 pack-years	453	6695	20	1.09 (0.68–1.74)	1.03 (0.64–1.66)
> 20 pack-years	1966	32,094	118	1.32 (1.03–1.70)	1.29 (0.70–4.98)
*p-value for trend*					*0.412*

*HR* hazard ratio, *CI* confidence interval ^a^Adjusted for age, no of births, Body Mass Index, physical activity, alcohol use, oral contraceptive use, age at menarche, menopause, age at 1st birth, parity, number of birth, hormone therapy use, and night shift work. ^b^21 is mean age at 1st childbirth in this cohort; ^**c**^ with information on age at cessation of menstrual bleeding; ^d^ every increase of 20 pack-years

No statistically significant interaction was found between smoking and menopausal status at the time of recruitment, BMI, HT, or alcohol use (Table 4).

**Table 4:** Effect modification of association between active smoking and breast cancer in 21,831 women in the Danish Nurse Cohort by menopausal status, obesity, alcohol consumption and hormone therapy use

			Never	Former	Current	Ever
	*N*	No. of cases	HR (95% CI)	HR (95% CI)	HR (95% CI)	HR (95% CI)
Menopausal status						
Premenopausal	9455	466	1.00	1.03 (0.82–1.29)	1.30 (1.04–1.61)	1.16 (0.95–1.40)
Postmenopausal	12,376	696	1.00	1.12 (1.92–1.37)	1.27 (1.05–1.52)	1.20 (1.02–1.42)
*p-value*					*0.77*	*0.80*
BMI						
Not obese (< 30 kg/m^2^)	20,578	1086	1.00	1.07 (0.92–1.25)	1.25 (1.08–1.45)	1.11 (1.03–1.33)
Obese (≥ 30 kg/m^2^)	1253	76	1.00	1.23 (0.72–2.09)	1.47 (0.81–2.66)	1.32 (0.82–2.12)
*p-value*					*0.84*	*0.63*
Alcohol Consumption						
None	3320	183	1.00	1.11 (0.77–1.61)	1.17 (0.83–1.66)	1.14 (0.85–1.54)
Moderate (1–14 drinks/week)	13,533	670	1.00	1.13 (0.94–1.37)	1.26 (1.05–1.52)	1.19 (1.02–1.40)
Heavy (>14 drinks/week)	4978	309	1.00	0.96 (0.70–1.31)	1.34 (1.01–1.77)	1.17 (0.90–1.52)
*p-value*					*0.66*	*0.99*
Hormone therapy use						
Never	15,880	755	1.00	1.05 (0.88–1.26)	1.21 (1.02–1.44)	1.13 (0.97–1.32)
Ever	5951	407	1.00	1.15 (0.88–1.49)	1.36 (1.06–1.73)	1 26 (1.01–1.58)
*p-value*					*0.63*	*0 33*

We found a slightly stronger association of smoking with ER+ (1.17; 1.01–1.36) than with ER- (1.11; 0.81–1.53) breast cancer, compared to never smoking (Table 5). When considering ER together with PR status, we found strongest associations of ever smoking with ER+/PR- breast cancer (1.75; 1.12–2.71), weaker with ER+/PR+ breast cancer (1.27; 1.02–1.59), and none with ER−/PR- (1.08; 0.72–1.611) or ER−/PR- (1.02; 0.26–4.07) breast cancer (Table 5).

**Table 5:** Association between ever vs. never smoking and breast cancer subtypes in 21,831 women from Danish Nurse Cohort

		Never	Former	Current	Ever
	*N*	Ref.	HR (95% CI)	HR (95% CI)	HR (95% CI)
Estrogen Receptor (ER) Status					
ER +	851	1.00	1.06 (0.89–1.26)	1.28 (1.09–1.51)	1.17 (1.01–1.36)
ER -	172	1.00	1.11 (0.77–1.62)	1.11 (0.77–1.61)	1.11 (0.81–1.53)
Receptor Status					
ER+/PR+	366	1.00	1.17 (0.90–1.53)	1.36 (1.06–1.76)	1.27 (1.02–1.59)
ER+/PR-	111	1.00	1.56 (0.94–2.59)	1.92 (1.19–3.10)	1.75 (1.12–2.71)
ER−/PR-	107	1.00	1.01 (0.63–1.63)	1.15 (0.73–1.82)	1.08 (0.72–1.61)
ER−/PR+	10	1.00	1.76 (0.41–7.56)	0.49 (0.08–3.01)	1.02 (0.26–4.07)

## Discussion

In this Danish Nationwide cohort of female nurses we found a positive association between active tobacco smoking and breast cancer incidence in women older than 44 years, with the highest risk in women who smoked heavily and for a long duration of time. The strongest risk was detected in parous women who smoked heavily before 1st childbirth.

Our results confirmed the association between active smoking and breast cancer incidence reported in almost all recent prospective cohort studies. [6–13] Albeit, the 27% increased breast cancer risk that we found in current as compared to never smokers, was stronger than the effects seen in any of the earlier prospective studies. In other previous studies the risk of breast cancer was reported to be increased by 24% in both the American Cancer Society’s Cancer Prevention Study II (CPS-II) [10] and the Women’s Health Initiative (WHI) [7] studies; by 19% in the Norwegian women [9]; by 15% in American retired women [13]; by 8% in American Black Women’s Health Study (BWHS) [8]; by 5% in American Nurses’ Health Study (NHS) [6]; and by 6% in the EPIC cohort. [11] We also found a strong effect of high smoking intensity, with a 32% increased risk of breast cancer risk in women smoking >20 pack-years; a risk comparable to 34% increased risk observed in Norwegian women smoking ≥16 pack-years, [9] but stronger than effects found in any other study. [6–8, 11, 13]

The strong effects of smoking observed in both, our and in the Norwegian cohorts (both that part of EPIC study [11] .and the other Norwegian cohorts [9]) may be explained by high prevalence, duration and intensity of smoking in these Nordic cohorts with 34% of the women being current smokers at the time of recruitment as compared with 13% in American nurses, [6] 8% in the American CPS-II study, [10] and 6% in the WHI study. [7] The average number of current smokers at the time of recruitment for the European countries in the EPIC study was 20%. [11] Furthermore, as we did not have data on passive smoking, our estimates for smoking are likely underestimated, as the EPIC study found that excluding passive smokers from the reference group led to higher risk estimates for active smoking. [11]

Notably, no association between smoking and breast cancer was found (current vs. never: 1.05; 0.72–1.54; former vs. never: 1.00; 0.68–1.47) in the Danish Diet, Cancer and Health cohort, which formed the Danish part of the EPIC study. [11] A possible reason for the difference between our results and those from Danish EPIC cohort could be that the women in the cohorts came from different birth cohorts. In the Danish EPIC cohort, the majority of women were born before 1944, and only 10% were born in 1945–1950. In the Danish Nurse Cohort, half of the women were born in 1945–1955 (Table 2). These younger birth cohorts started smoking earlier and were able to accumulate more years of smoking before the 1st childbirth than the older birth cohorts: on average 7.8 years as compared to 5.3 years (Table 2). The oldest generations in the Danish Nurse Cohort furthermore included a high proportion of nulliparous women; 27% for nurses born in 1990–1934 (Table 2), reflecting that before the Second World War Danish nurses were mostly unmarried. Finally, the EPIC cohort might be affected by a “healthy worker effect”, as the cohort members were recruited from general population and only 37% participated [23] as compared to 86% participation rate in the Danish Nurse Cohort. [18] While the percentage of never smokers was 36% in the Danish Nurse Cohort, it was 43% in the Danish EPIC cohort. [11]

We found the highest risk of breast cancer related to smoking heavily before the 1st childbirth, in agreement with existing evidence. [6–8, 10–12] Breast development with increased epithelial cell proliferation begins before the menarche, [24] while the terminal differentiation of the breast epithelium takes place in the last trimester of pregnancy. [25] It is therefore plausible that the time before the 1st pregnancy may be critical for susceptibility to tobacco smoke carcinogens. In contrast to the EPIC study, [11] no protective effect of smoking in postmenopausal age was found in either American [6] or Danish nurses.

As alcohol is an established risk factor for breast cancer, and as alcohol and smoking often come together, the possible confounding by alcohol of the effect of smoking on the risk of breast cancer has been debated in literature. Alcohol has also been previously shown to be an independent risk factor for breast cancer in the Danish Nurse Cohort. [26] However, alcohol did not seem to be a confounder in our study. Our risk estimates were adjusted for intensity of alcohol consumption. The breast cancer risk was somewhat enhanced in heavy drinking current smokers, but, in line with the results of the EPIC study, [11] the trend of increase in risk of smoking by increasing level of alcohol consumption was not statistically significant neither for ever nor for current smokers (Table 4). The available data thus indicate that the effect of smoking on breast cancer risk is independent of the effect of alcohol.

We contribute with novel data on smoking and subtypes of breast cancer, as only a few studies have previously included this information. [7, 10, 11, 13] We detected a very strong association between current (vs. never) smoking and ER+/PR- breast cancer (1.92; 1.19–3.10), followed by a weaker but robust association with ER+/PR+ breast cancer (1.36; 1.06–1.76), and none for ER−/PR- or ER−/PR+ breast cancers. This is in agreement with EPIC study, which reported the strongest risk related to current smoking of 34% for ER+/PR-, 23% for ER+/PR+, and 13% for ER−/PR-, [11] and American Retired women study which found 37% increased risk of ER+/PR- breast cancer in current smokers, and none with ER+/PR+ or ER−/PR- subtypes. [13] Luo et al. has reported a 28% increased risk of ER+/PR+ breast cancer in current compared to never smoker, but in contrast to our study and current literature, none with ER+/PR- or ER−/PR-, [7] whereas Gaudet et al. reported association with smoking limited to ER+ breast cancer subtype, and none with ER-, but lacked data on PR status. [10]

Strengths of this study include data from a large prospective nationwide cohort with comprehensive follow-up of both vital status and incident breast cancer cases from linkage with nationwide registers. Exposure information from the cohort was expected to be valid as already known associations between breast cancer and, for instance, alcohol use [26] and HT [26–28] have already been documented in this cohort. Another strength was the high smoking prevalence, where 34% of women in our cohort were current smokers. This means that potential confounding not controlled for will affect our results less than is the case in the studies where smokers constitute a smaller, and thus a more marginalized group of women.

The main limitation was the exposure misclassification as the smoking exposure was based on questionnaires at the time of recruitment, without follow-up, and the lack of information on passive tobacco smoke. However, prevalence of active and passive smoking in Denmark in this period was high, and based on data from a related Danish Diet, Cancer and Health cohort recruited in the same period, we estimated earlier that only 5% of women in this period were not exposed to passive smoke at home or work. [29] In any case, passive smoking is difficult to assess accurately, due to its ubiquitous exposure, and the American nurses study failed to find an association between passive smoking and breast cancer, [6] while the EPIC study found that excluding passive smokers from the reference group led to higher risk estimates for active smoking. [11]

Danish nurses have been found to live a generally healthier lifestyle than a representative sample of Danish women, as they smoked less and had higher physical activity levels, although they consumed more alcohol. [18] Furthermore, there was no major differences between Danish nurses and Danish women in general with respect to use of health care and disease occurrence. [18] It is therefore reasonable to generalize the findings based on the Danish Nurses Cohort to Danish women in general.

## Conclusion

In this cohort of Danish nurses, we found the risk of breast cancer to be almost 30% increased in current smokers as compared with never smokers. The risk increases both with smoking duration and smoking intensity. The highest risk was seen in women with more than 10 pack-years of smoking before the birth of their first child. We found no protective effect of smoking in post-menopausal age. The study contributed to the accumulating evidence for smoking - in particular in early life – as causally associated with an increased risk of breast cancer.

## Abbreviations

BMI: Body Mass Index
BWHS: Black Women’s Health Study
CI: Confidence Intervals
CPS-II: Cancer Prevention Study II
EPIC: European Prospective investigation into Cancer and Nutrition
ER: Estrogen Receptor
HR: Hazard Ratio
HT: Hormone Therapy
IARC: International Agency for Research on Cancer
OC: Oral Contraceptives
PR: Progesterone Receptor
WHI: Women’s Health Initiative
